# Clinical Understanding of Spasticity: Implications for Practice

**DOI:** 10.1155/2014/279175

**Published:** 2014-09-04

**Authors:** Rozina Bhimani, Lisa Anderson

**Affiliations:** ^1^Department of Nursing, St. Catherine University, St. Paul, MN 55105, USA; ^2^Department of Integrative Biology and Physiology, University of Minnesota, Minneapolis, MN 55455, USA

## Abstract

Spasticity is a poorly understood phenomenon. The aim of this paper is to understand the effect of spasticity on daily life and identify bedside strategies that enhance patient's function and improve comfort. Spasticity and clonus result from an upper motor neuron lesion that disinhibits the tendon stretch reflex; however, they are differentiated in the fact that spasticity results in a velocity dependent tightness of muscle whereas clonus results in uncontrollable jerks of the muscle. Clinical strategies that address function and comfort are paramount. This is a secondary content analysis using a qualitative research design. Adults experiencing spasticity associated with neuromuscular disorder were asked to participate during inpatient acute rehabilitation. They were asked to complete a semistructured interview to explain and describe the nature of their experienced spasticity on daily basis. Spasticity affects activities of daily living, function, and mobility. Undertreated spasticity can lead to pain, immobility, and risk of falls. There were missed opportunities to adequately care for patients with spasticity. Bedside care strategies identified by patients with spasticity are outlined. Uses of alternative therapies in conjunction with medications are needed to better manage spasticity. Patient reports on spasticity are important and should be part of clinical evaluation and practice.

## 1. Introduction

Disability can have devastating effects on people's lives. It is estimated that about 15% of people in the world live with different types of disabilities [1]. Among these disabilities, physical disability due to neuromuscular dysfunctions (such as spinal cord injury, multiple sclerosis, stroke, and traumatic brain injury) is particularly devastating because neuromuscular dysfunction can lead to immobility, social isolation, pressure ulcers, increased urinary tract infections, and other sequelae due to primary disability [1]. Spasticity is one of the sequelae of neuromuscular disability [2]. Martin et al. [3] estimate prevalence of lower limb spasticity for stroke (40–600 per 100,000), multiple sclerosis (2–350 per 100,000), cerebral palsy (260–340 per 100,000), and spinal cord injury (22–90 per 100,000). They estimate annual incidence of lower limb spasticity for stroke to be 30–485 per 100,000, traumatic brain injury 100–235 per 100,000, and spinal cord injury 0.2–8 per 100,000.

The most common understanding of spasticity has roots in Lance's study [4]. In this study, spread of phasic reflexes in four normal subjects and sixteen patients experiencing spasticity was evaluated through electromyography. Later this study provided the basis for the spasticity definition [5], which is now commonly available in the literature. There is plenty of research available on spasticity; however, patients' feedback on their understanding of spasticity is missing from the literature. Spasticity symptom experiences can be devastating and patients' spasticity interpretation may differ. Ethical clinical practices require clinicians to incorporate patients' understanding of this phenomenon in their plan of care. Bhimani et al.'s [6] original study reports on the patient understanding of spasticity and their results indicate that there is a discrepancy between patients and clinicians understanding of spasticity. Therefore, omitting patient reports from clinical decision making can have grave and serious consequences on their lives manifesting as side-effects of spasticity therapy, administration of invasive and inappropriate therapies, unnecessary pain, and suffering.

## 2. Background

### 2.1. Movement and Posture Physiology

One of the essential functions of the human motor system is to determine the correct joint positions and movements, specifically, a motor program, required to carry out the daily activities of life. In addition, a motor system must integrate the motor intention of the individual with the muscle tone and body position information from the musculoskeletal system to determine that motor program. Muscle tone refers to continuous partial resistance that prevents full relaxation of voluntary muscles. This resistance is felt due to the elasticity and compliance of the tissue when a limb is moved through passive range of motion [7]. This partial tension in the muscle is needed for a muscle to act quickly and smoothly to carry out a future task. For example, an act of throwing a ball contracts the bicep muscles while the triceps muscles also cocontract to stabilize the elbow joint during this movement. On the other hand, the act of tapping the bicep tendon causes the bicep muscles to contract (increase in muscle tone: an agonist act) while the triceps muscle relaxes (decrease in muscle tone: an antagonist act) at the same time to maintain muscle length. This change in muscle action is mediated by a monosynaptic reflex arc through spinal cord of the central nervous system (CNS). This type of normal reflex function is known as the tonic stretch reflex (TSR). The connection of the CNS with muscles further provides reflex activity to maintain balance and posture in the body. The stretch reflex is a negative feedback mechanism that maintains muscle length; compliance is an intrinsic property of the muscle and connective tissue [8].

**Figure 1:**
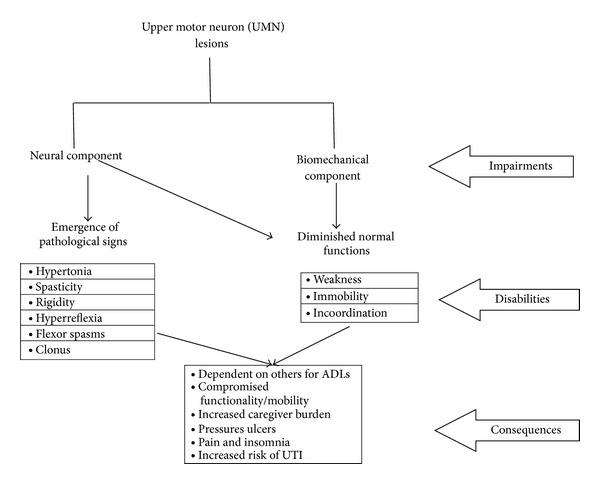
Upper motor neuron (UMN) syndrome impairment, disabilities, and its consequences.

Homeostasis of normal muscle tone is disrupted for many reasons. In some instances, pathways to maintain muscle tone are disrupted due to injury-producing lesions in motor neurons. Neurons that connect the brain to the spinal cord are known as upper motor neurons (UMNs), whereas neurons that connect the spinal cord to muscles are called lower motor neurons (LMNs). From the brain, upper motor neurons provide the movement instructions transmitted through the lower motor neuron to the muscles. When the lower motor neurons are damaged, the result is twitching of contiguous fibers (fasciculation), muscle weakness, and atrophy [9]. When the UMNs are impaired, information needed to maintain a normal TSR is disrupted, leading to the pathologies of muscle tone that create spasticity, which can be permanent (see Figure 1). The consequences of these pathologies may result in dependency in activities of daily living (ADL) and mobility, create pressure ulcers, and increase caregiver burden [10].

## 3. Review of the Literature

This is a critical review of the literature that clarifies neurological disorder of spasticity, hypertonia, and related terms. Following types of hypertonia are clarified by outlining the pathophysiology for spasticity and clonus. Neurological terms such as spasticity and hypertonia and associated terms require clear understanding and interpretation in clinical practice for safe patient care.

## 4. Hypertonia

Hypertonia is an “umbrella term” that describes any condition leading to tight or stiff muscles. Many clinicians use the term hypertonia interchangeably with spasticity; however, spasticity is a type of hypertonia that is velocity dependent or in other words is increased with movement, though spasticity can be present at rest. In clinical practice, spasticity is often confused with other conditions such as clonus and rigidity. The velocity dependent aspect of spasticity distinguishes it from rigidity, which is not velocity dependent. Rigidity is a symptom seen with basal nuclei lesions, such as Parkinson's disease. With rigidity, muscles exhibit the same degree of tightness regardless of the amount of movement or sensory input [11]. Spasticity as a sensory-motor disorder acknowledges that sensory stimuli are an influence on the experience of worsening spasticity. Clonus is also distinguished from spasticity; clonus is involuntary jerks and tremors of the limb. It can concurrently be present with spasticity and rigidity.

### 4.1. Upper Motor Neuron

The tight, stiff muscle experienced with hypertonia is a symptom of upper motor neuron dysfunction. Nerve impulses are normally relayed from the UMN to interneurons in the spinal cord and then through the LMN to muscles [8]; UMN damage and the loss of this relay information can impact functions such as walking, breathing, and swallowing. Disruptions in these functional movements can compromise activities of daily living and even can be life threatening. Tight muscles can cause pain and disrupt sleep at night. The increased muscle tone can have a devastating effect on the quality of life. Tight and rigid muscles can result in contractures that affect ambulation, ADLs, comfort, and sleep [10, 12]. Therefore, upper motor neuron dysfunctions demand the full array of therapeutic treatments and interventions [13].

## 5. Pathophysiology of Spasticity

**Figure 2:**
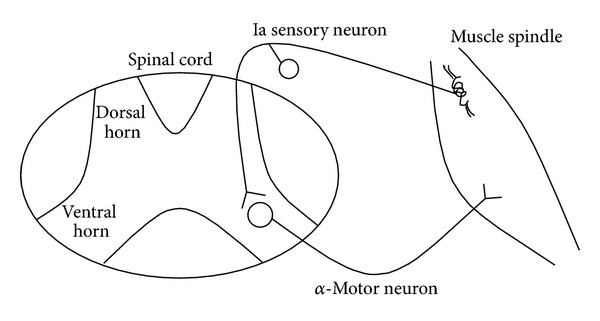
Basic monosynaptic stretch reflex.

Spasticity is a part of a disabling UMN syndrome. The UMN lesions decrease the inhibitory drive in the corticospinal tract to produce spasticity. Spasticity is generated through local activation of muscle spindles, but the propagation and manifestation of spasticity require involvement of the central nervous system [2, 14]. The UMN lesion disrupts communication between the brain and the spinal cord, producing a state of net disinhibition of the spinal reflexes. In spasticity, when a limb muscle is stretched muscle spindles respond by sending action potentials to the spinal cord via sensory neurons. However, the negative feedback system between muscle spindles and alpha-motor neurons is disrupted because of the UMN lesion, and abnormal muscle activation occurs. There are many interrelated feedback mechanisms that can account for spasticity [15, 16]. For example, the UMN lesions decrease the inhibitory drive in the corticospinal tract, which can affect alpha-motor neuron excitability causing increased muscle contraction, particularly in flexor muscles. Furthermore, motor tract that originates in the brainstem can increase excitation of spinal neurons [15, 16] (see Figure 2). Also disruption of interneuron mediated inhibition of the antagonist muscle or increased action potentials in the sensory neurons from the muscle spindle can lead to hypertonia [15, 16].

### 5.1. Sensory Experiences

Spasticity is a sensory-motor phenomenon. The new definition from the Support Program for Assembly of a Database for Spasticity Measurement (SPASM) project defines spasticity as “disordered sensory-motor control, resulting from an upper motor neuron lesion” [17, page 72]. Involvements of both the sensory input and motor outputs are identified in producing spasticity. Currently clinical practice is still based on the narrow Lance [5] definition of spasticity. Lance [5] defined spasticity as a “motor disorder characterized by a velocity-dependent increase in tonic stretch reflexes (muscle tone) with exaggerated tendon jerks, resulting from hyperexcitability of the stretch reflex” (page 485). This definition is useful in clinical practice, because, by providing the guideline of “velocity-dependent increase in tonic stretch reflexes,” it delineates spasticity from other similar movement disorders such as hypertonia, rigidity, and hyperreflexia. Unfortunately, this ignores the important aspect of sensory input in the experience of spasticity. Lance's [5] definition is therefore limiting and somewhat misleading [6]. Therefore, it is imperative that clinical practice incorporates the new 21st century definition to operationalize the management of spasticity.

## 6. Pathophysiology of Clonus

The pathophysiology of spasticity and clonus is separate but interrelated. Clonus is generally more easily induced in the ankles and feet. When the ankle is dorsiflexed, muscle spindles are stretched in the gastrocnemius muscle which is sensed by sensory receptor Ia and impulses are sent to the spinal cord where it synapses on the alpha-motor neurons leading to more activation of alpha neurons to the gastrocnemius and soleus. Contraction of the gastrocnemius and soleus leads to plantarflexion. Cycles of plantarflexion and dorsiflexion follow as the gastrocnemius and soleus contract quickly and then relax and stretch in a repeating loop [18]. Competing explanations for the ankle clonus are found in the literature. Though initially ankle clonus was thought to be the oscillations of antagonistic muscles, later EMG studies show that this is not the case. During ankle clonus, strong EMG activity is observed in the gastrocnemius and soleus but not in the anterior tibialis, the antagonistic dorsiflexor [18]. Ankle clonus was therefore hypothesized to be an exaggerated stretch reflex of the gastrocnemius and soleus only. Other investigators have observed EMG activity in the anterior tibialis, occurring simultaneously with EMG activity in the plantarflexors [19, 20], leading to the hypothesis that a central pattern generator mechanism in the spinal cord was at least jointly responsible for clonus. The observation that cold therapy significantly decreases clonus, whereas the central alpha2 antagonist, tizanidine, does not, suggests that peripheral mechanism is more important [21], though the mechanism is still being debated.

With an UMN lesion leading to spasticity and clonus, alpha neurons are activated too much with the loss of inhibition from corticospinal and dorsal reticulospinal corticobulbar pathway and excitation from vestibulospinal and medial reticulospinal pathways [16]. Highly activated alpha neurons increase the sensitivity of muscle spindles, making hypertonic spasticity and the repeating contractions of clonus possible [16]. Furthermore, interneuron mediated relaxation of antagonist muscles is prevented, adding to more contraction and oscillation of impulses [16, 22].

## 7. Quality of Life Issues

Spasticity and its consequences negatively influence quality of life. Management of spasticity is an important care issue in neuroscience because it can affect quality of life. Very few first-person accounts of spasticity are available. Most literature has focused on the quantification of symptoms but patient's input is lacking [13, 23, 24].

Despite the fact that spasticity is a lived experience, there are very few studies on patient accounts and perception of spasticity. Bhimani et al. [6] and Mahoney et al. [25] have explored overall understanding of spasticity. Both report similar findings that patients understanding of spasticity was based on the individual interpretations and meaning assigned to the experience. Symptom experiences of spasticity were either accepted or managed medically or with use of alternative therapies. Social consequences of spasticity experiences were embarrassment and social stigma, which led to self-isolation. In addition, participants reported sensory experiences and found themselves unable to articulate these sensations. Patients often used word spasm to report both clonus and spasticity.

Spasticity is a fertile area for research. Clinical outcome measures to assess spasticity at the bedside cannot completely account for the myriads of spasticity experiences. Understanding of spasticity and clonus from patient perspective and how it affects daily life is needed for clinical understanding and bedside practice.

## 8. Method

The purpose of this study is to understand the effect of spasticity and clonus on daily life and identify bedside strategies that enhance patient's function and improve comfort.

### 8.1. Research Question

What are spastic patient experiences of activities of daily living, function, mobility, comfort, and pain?

Specific aims were

1. to identify missed opportunities for care during inpatient rehabilitation stay;
2. to identify bedside strategies for care in practice.

### 8.2. Design

This is a secondary analysis of the original longitudinal qualitative study to identify strategies that impact spastic patient's activities of daily living, mobility, and comfort.

### 8.3. Sample

The study setting was a 31-bed acute rehabilitation unit in a large nonprofit tertiary-care hospital and a stand-alone rehabilitation center with 56 beds both located in a metropolitan area of the Midwestern United States. Both settings were at Commission on Accreditation of Rehabilitation Facilities (CARF) certified for spinal cord injury (SCI). Administrative and institutional review board permissions were obtained from both facilities. A total of 23 participants were recruited with neurological disorders associated with spasticity. Out of 23 participants, the first 10 participants were recruited from the hospital-based rehabilitation unit. The remaining 13 participants were recruited from the stand-alone center. The majority of patients were Caucasian (83%), with the diagnoses of spinal cord injury (61%), stroke (17%), cerebral palsy (13%), and multiple sclerosis (9%). About equal number of men (48%) and women (52%) were recruited, with a majority of participants (57%) under the age of 45 and the rest were under the age of 65. Neurological deficits included inability to perform ADLs independently (100%) and mobility impairments where 22% needed some assistive device while 78% were wheelchair bound, of which 13% could not move upper or lower extremities. All participants' hearing, vision, and speech were preserved.

### 8.4. Procedure and Data Analysis

The original research study focused on understanding spasticity over time. Subjective understanding of spasticity was extracted from responses to a daily open-ended question about spasticity. The purpose was to allow patients to freely express their spasticity experiences nested in daily routines. In addition to daily open-ended question, twice a week patients were asked in-depth questions to capture changes in spasticity through words during their routines of daily living. Participants were followed up for seven days in the inpatient rehabilitation setting.

During data analysis for the original study, it was apparent that the participants provided large amounts of data. Therefore, a secondary data analysis was undertaken guided by the current research question. Colaizzi's method [26, 27] guided the secondary analysis. Initially, transcripts were read in their entirety to get the sense of the content. During this phase, interesting areas of the content for patient daily routines, mobility, comfort, and nursing interventions were noted using memos. These were emerging codes. Careful attempt was made to assure that the context was carried through these emerging codes. Refinement of this organized data led to categories reflecting the most descriptive topics in all or most of the data. Then these categories were viewed for their content to make sure that they captured the category. Here, similarities and uniqueness in content along with confusion and contraindications were identified. Content analysis for this data continued until discrepancies were resolved and themes emerged.

Lincoln and Guba's [28] criteria for qualitative paradigm guided the rigor in the study. Keeping in mind the rigor for qualitative inquiry, the researcher kept reflective journaling to identify her preconceived notions and biases. Audit trails for content analysis were reviewed several times to enhance credibility. Conformability was achieved by evaluating separate data chunks at different times. These steps further enhanced dependability, transformability, and authenticity.

## 9. Results

### 9.1. Activities of Daily Living

Experiences of spasticity were embedded in the circadian rhythm. Spasticity experiences were not bounded by the days or hours. Instead, the best clues for understanding spasticity may be understood through rhythms of one's biological clocks and daily activities of life habits based on the institutional routine during acute rehabilitation. At a glance, spasticity may appear to be unreliable and unpredictable; however, closer look reveals that experiences of spasticity appear to be linked with activities of daily rhythm, as these participants become cognizant of how certain things affect their symptoms of spasticity.

*In the morning when the aides are turning me over, when they turn me, they have to turn me so many hours every night. Those are when I'm going through my worst spasms then in the middle of the night and late in the afternoon. #101*

Patients' spasticity fluctuated throughout the day. Individual participants reported that their life revolved around activities of daily living (ADL). Most of them spent considerable amount of time dealing with their routines. ADL routines either in the morning or at bedtime require considerable energy and participants often felt frustrated because the body did not respond on command.

*Spasticity should be, I think be termed as anything that any involuntary muscle movement that interferes with a person's daily living and that is the exact opposite of what they tell their body to do. #201*

*It made it difficult for dressing, getting up and all that stuff, that's all I know, that's the big change. I can't go anywhere I want, because I just can't get up and go, that's my change area. I can't normally do what I normally do. #141*

Since increase in clonus and spasticity activity may be triggered by simple touch, patients felt helpless and troubled. Some participants in the study reported being afraid that their condition might cause an injury to their caregivers during ADL.

*I'm always afraid I will kick whoever getting me up and stuff. #151*

* Like last night I told the RA (assistant) to get out of the way and zap I was kicking and waving on my bed and she was out of the way. She said you almost kicked me. I replied, Yes, but I gave you lead time, you know not to come close to me when I'm having a spasm (clonus). I don't think anybody is ever going to understand that unless, well maybe in 500 years. # 181*

*Missed Opportunity*. Most participants wanted their caregivers to slow down while assisting them with ADLs. Often they felt that caregivers were so engaged in “tasks” that they lack patience with them. They felt rushed in their care which compromised their sense of control over ADLs.

*A little more patience. I think they lack a lot of patience. Some of them, not all of them. You have some who come in that just lack patience with the patients that are here. #191*

*A lot of times they seem like they're in a rush and a lot of them like say, for shaving, cleaning their ears out or whatever it is, you know stuff like that that is an every-day thing. #171*

### 9.2. Function and Mobility

Physical cost of spasticity is paramount as mobility limitations and limb impairments reduce their chances to fully engage in their lives. Spasticity patients struggle with mobility, function, and transfer issues. Although some participants had families and jobs, they were dependent on others for their daily routine care. This dependency on others can diminish their quality of life. Mobility impairments leading to possible falls are a genuine concern. Gait instability or violent tremors can contribute to the risk of fall. Fear of falling is a genuine worry because muscles do not respond on command.

*Just that it's an out of control feeling. Things kind of jerk up and tighten up a little bit. If I didn't have seat on my chair, I'd probably be laying on the ground. #151*

Use of a wheelchair did not always make them feel secure; often issues with body positioning relative to the chair were a concern as participants reported.

*I also think that the way that your chair is made effects that. Because if you can move around and put your legs in a different position when you're feeling tense, I think that would help a lot. #161*

*I don't know, because I'm sitting upright all the time and I'm not laying back. The more lay back the more my spasms kick in. If I'm sitting up, they're not bad, if I lay back, they're bad. #131*

*Missed Opportunity.* Common concerns reported by patient participants were that their caregiver did not understand their mobility restrictions. Just because they were in a wheelchair, they did not perceive themselves mobile as given that spasticity affected their entire body.

*I think they either should, like say like somebody ain't got the use of their hands and that they should after the aide get done with you, they should help you down to the elevator you got to go down. Like me, I can't push the buttons or stuff like that I think they should follow them down. Right now what I'm doing I'm going myself and then just seeing if there's somebody there to, I'm asking people walking by to turn the elevator on. #191*

### 9.3. Pain, Comfort, Insomnia, and Fatigue: A Vicious Cycle

Pain and insomnia are intricately linked in this population due to muscle tightness. The patients experiencing clonus may have sleep issues as constant jerking motion may not allow them to fall asleep easily. On the other hand, spasticity patients simply cannot relax due to tight muscles. Therefore, aggressive management of the troubling symptoms is needed to improve quality of life. This uncomfortable symptom experience of insomnia can lead to fatigue, which in turn creates stress and further increases spasticity. Therefore, caregivers must be cognizant of all these interrelated clusters of symptoms and address these issues in a comprehensive manner.

*During later in the day they (clonus) too get more frequent so I guess that's about all on that one… Oh, sometimes they wake me up. Then I have to get repositioned and get some ice. #131*

*There's nothing else you can think about but the pain that's going through your arms and through your legs, there's not too much you can do about it. # 191*

*If muscles are incredibly tight and I haven't stretched at all, I do experience quite a bit of pain… I'm always tired so I have to really concentrate in order to get my body to do anything that I want it to do. #201*

*Right now I'm in a real bad sharp pain radius and it's really killing me. It's really testing me and I just want to go somewhere and bark I'm in pain right now. #141*

*We were sitting by the parking lot in the shade and I had quite a few (clonus). I decided to go in because I was drained, I couldn't even drive (wheelchair) I was drained so much. Like I'd run a marathon. #181*

The most prominent finding of this study relates to the impact of daily stressors on the spasticity experiences. Most patients were very vocal about the negative impact of stress from emotions such as being angry, feeling rushed, and experiencing anxiety. To combat this, some participants took refuge in meditation, acupuncture, and prayers.

*Once I get stressed or if there is a lot of stuff going on, my tone will automatically increase. #81*

*I like to talk on the phone, it relaxes me… usually I talk to my parents and my friends, like today I'm going to talk to this friend of mine that I haven't talked to in a long time. #161*

*Where they have this little Chinese herbal medicines or needles in different parts of your body this is an energy movement or guided imagery or things like that. #151*

*Missed Opportunity.* Most spastic patients require stretching throughout the day to manage spasticity. If not done with frequent intervals, it led to suffering and pain.

*So if you don't move then you come out and try to move it ain't going to work. I mean the muscles are all this cramped up. That's why they had a hard time trying to stretch me out I was screaming a lot, because all the muscles were all the way you left me lay bent up so try to straighten something when you're bent, laying in there like that, that's all I can say on that. #141*

### 9.4. Other Factors

Comorbidities also seemed to affect spasticity. One participant was experiencing urinary tract infection and a kidney stone. There was an increase in clonus activity where spontaneous series of uncontrollable tremors were noted without provocation. Spasticity was noted to increase as well.

*I started realizing I had good days and I had bad days and when I really noticed it is when I would get a UTI and then my spasticity would be terrible and I would be so uncomfortable. #81*

*I do have a UTI now, an upper urinary tract infection and that, in me, can set off spasms (clonus). I'm prone to colonizing if they put me on antibiotics. I was worried about that on the last week because my UTI wasn't clearing up. But they're managing it with something else and I drank a couple of full water bottles during the day, which makes me have to go and I think it's clearing it out is what's happening. #181*

*Missed Opportunity.* Clinician often used vocabularies that patient did not understand. One participant did not know that the “complete” and “incomplete” spinal cord injury have different connotation in medical jargon.

*I was thinking it meant that the nerves is there (partial and incomplete SCI) and they going to connect, if you strong enough, they going to start connecting to each other, that was what I was thinking it was, connecting to each other and if I got the strength to do it, it will come together and you will be ok, I got to have the strength and the willpower and I got to make it happen and I can't make it happen…That ain't true, it don't work that way, it do not work that way. #111*

### 9.5. Bedside Care Strategies

#### 9.5.1. Medications and Other Modalities

Spasticity is managed by physical therapy, drugs, and surgery. Oral antispasmodic medications such as baclofen, tizanidine, and dantrium are used for initial management of spasticity and clonus while intrathecal baclofen may be used for severe symptom management [29, 30]. Usefulness of oral medications may be limited due to the side-effects such as hepatic toxicity, fatigue, drowsiness, weakness, and nausea. Our participants provided some guidance to clinicians. Although most felt medications were needed, they felt that approach to spasticity management must be holistic.

*To sum it all up, I think doctors should give their patients options and strategies for coping with spasticity based on what the patient says versus what is happening clinically. #201*

*I think there should also be a more holistic approach like deep breathing and stuff like that seems to help me and like being in warm water. Right now it's more of a clinical, what can we do with drugs to treat this condition mindset. I think a lot more can be done with natural medicine and that's what I'm hoping anyway. #71*

#### 9.5.2. Stretching

Range of motion and stretching is a corner stone of spasticity management. Since muscle tightness causes pain and contractures, most participants were on scheduled physical therapy and stretching routines. Some stretching was done as a part of activities of daily living while intentional stretching was embedded throughout the day. Participants felt that this care strategy was particularly important to maintain comfort and function.

*They are laying there, my left leg I can feel more in that leg than in the right leg. It feels so stiff so when they move them I can feel the pain in it when they start doing the exercises. They are supposed to do it every so often when I'm lying down, especially early in the morning and before I go to bed at night but I don't know what kind of schedule they have here. Every schedule is always different, you know when the aide comes in so this morning she did a little bit of it, but I don't think she did enough of it.*

*Especially in the morning I like to stretch and it takes me a while to get up or feel like I want to get up and get moving because I like to stretch. #151*

*It's a lot different when I'm going to physical therapy that they stretch a lot better than the aides do it. My leg feels a lot better, I can feel the tightness going away, which the aides do it, they don't, the tightness is still there when I get down to therapy and I can feel it in my legs as I'm going down there. #91*

#### 9.5.3. Comfort Strategies

Over time participants had figured how to self-manage their symptoms of spasticity and clonus. They shared the fact that keeping sudden noise and touch to minimum is important in preventing inducement of clonus and spasticity. Pool therapy with warm water was desired and seen as a good relaxation therapy.

*I think the water just holds you up so you don't have to concentrate. They have had things that hold your head up so you barely have to do anything so the muscles completely relax, that's when I would say I am the least spastic or when I am in any type of water, I'm a lot more relaxed than normally. #161*

*“What makes it better is generally environmental factors that just hot and cold and not having too much going on.” #31*

Most participants identified warm temperature helpful in management of spasticity; however, one participant reported use of ice at night time to facilitate sleep.

*Lately, I've been waking up in the morning with severely cramped arms to the point I couldn't move again. Not this morning, every morning for about a week prior, I've been waking up with arms just clenched to my chest and I couldn't move them until I got range in the morning and then they seemed to loosen up a little bit. One thing I did different last night when I didn't wake up this morning with the cramped arms, was I wore a sweatshirt to bed. I was hot sleeping, the room is cool, air conditioned, it was cold outside last night and I wore my sweatshirt to bed and it kept my upper torso warm and I woke up with basically no cramping this morning so I'm going to keep doing that from now on. #181*

*The cold pack does help at night. #121*

*A loud noise you know something like that…I'm going to jump now, I think that would change if I didn't have so much spasticity. #30*

#### 9.5.4. Mood and Alternative Medicine

Feeling negative or depressed was reported to increase spasticity. Most participants acknowledge mind-body connections and felt that their care strategies should include more than “western medicine” approach. Participants shared utilization of alternative medicine in conjunction with western medications.

*If I'm relaxed, I'm happy, if I'm upset, I'm more tense, at least for me. Mood really changes it for me. I think when you're more relaxed, it's different. I think more people should, or the doctors should recommend acupuncture or something for relaxation, I don't know if I would do it but if I had a doctor's order, I might. My roommate does it and she really likes it, she's like you should try it. #161*

*We're going to waste a lot of money by going backwards instead of trying to go forward and try some of their natural herbs and the way they do the acupuncture, it's been helping a lot of other people. And, they've been doing it a long time before we even thought about it. #20*

*My mother-in-law is teaching me meditation and I'm going hypnotism too at the same time. Those two things with the medication is kind of keeping me in save. That's where I am right now. Those three things is going on and my family. My family is a big help. #111*

#### 9.5.5. Individualize Care

Most patients considered their understanding to be unique and individual, as the essence of spasticity was learned through personal accumulated experiences over time. There was a strong sense that, to understand spasticity, one must live it to understand the pain and suffering.

*I don't think anybody is ever going to understand that unless, well maybe in 500 years, you can't live inside another person's body, you don't know what you're going through and it's very difficult to explain to other people… The only to experience it is to have to happen to you, I think. #181*

*Someone who hasn't experienced severe spasticity has no frame of reference so their perception of their spasticity may be more severe than the clinician thinks… in my opinion, it would be very important for doctors to look at a patient based on what their baseline is as far as spasticity. #201*

## 10. Discussion

Research provides the guidance for clinical issues in practice. Qualitative researchers are required to understand human meaning associated with a phenomenon to provide better health care experiences. As Van Manen [31] pointed,

*to establish a strong relation with a certain question, phenomenon, or notion, the researcher cannot afford to adopt an attitude of so-called scientific disinterestedness. To be oriented to an object means that we are animated by the object in a full and human sense. To be strong in our orientation means that we will not settle for superficialities and falsities* (Page 33)*.*

The understanding of a phenomenon is socially, temporally, and contextually bound and dependent on the individual's realities. The essence of a phenomenon may be universal but its varied interpretation provides some degree of depth and richness of those experiences. Interpretation is fluid, based on context and temporal nature of the experiences. Van Manen [31] further adds,

*the “data” of human science research are human experiences. It seems natural, therefore, that if we wish to investigate the nature of a certain experience or phenomenon, the most straight forward way to go about our research is to ask selected individuals to write their experiences down* (Page 63)*.*

The results of this study indicate that spasticity is a lived experience. The richness of this lived experience captures its essence in the context of health. The lived experience of spasticity calls for the eudemonistic approach of well-being. This incorporation of patient accounts captures the essence of their symptom experiences, which in turn assists clinicians in matching their practice with patient experiences. This clarity and congruency have implications for the quality of life for patients who suffer from spasticity. Therefore, this study provides glimpses into the lives of spastic patients through their own lived experiences. Although researchers and clinicians want accurate definitions, precise measurement tools, and state-of-the-art interventions to help human suffering, individuals who are suffering from spasticity, their input is critical for evidence-based practice. The clinicians face many challenges when caring for spasticity patients as this population experiences myriad of sensations due to neurological deficits. Some patients may be experiencing L'Hermittes sign, which could be part of the multiple sclerosis or spinal cord injury experience, where patients describe a sensation of electric shock going through their body [32]. Others may describe sensation of pain with spasticity. In this population, most clinicians associate pain with neuropathic pain from neurological disorders [16]. However, if the patient is experiencing spasticity, one must take the time to assess the entirety of the situation to understand whether pain is due to spasticity, development of neuropathic pain, or something else. Correct evaluation of spasticity symptoms is imperative in providing safe, effective, and individualized care with compassion.

### 10.1. Implication for Practice

Spasticity is often accompanied by uncomfortable pain, which may be due to the nerve entrapment in the muscles. Untreated spasticity can lead to contractures. Subsequently, pain, pain-induced insomnia, contractures, mobility impairment, potential for falls, and development of pressure ulcers due to tight muscles over bony prominences can impact quality of life.

Bedside interventions such as avoiding inactivity and utilizing warm pool therapy may be helpful. Our findings support Boyraz et al. [21] that cold temperature may be helpful for some patients experiencing clonus. Most of these patients may benefit from stretching and range of motion exercises in maintaining comfort, posture, and movement. Medications are important in managing their symptoms on a daily basis as it provides comfort and relaxes their muscles enough so that they remain functional. The other important interventions are the use of physical and occupational therapies [6]. The participants in our study reported use of alternative therapies such as acupuncture, meditation, and prayers being helpful in decreasing spasticity. Their feedback and suggestions provide opportunity to explore the role of alternative therapies in managing spasticity.

Mobility impairments leading to possible falls are a genuine concern. Even though risk of falls is an obvious issue, fall risk assessments are not routinely employed in an outpatient clinical practice. Since it is clear that an inherent risk for falls is present in this population, routine formal assessment of this risk should be done so that clinicians can assess patients status and their environment to minimize this hazard.

The experience of spasticity is a quality of life issue, as its effects are pervasive in all domains of life. Therefore, clinicians must be cognizant of the consequences of this experience and mobilize appropriate resources based on individual needs. Individuals not only need medical intervention but also may require psychological counseling and strong spiritual connections to find an acceptable quality of life.

## 11. Conclusion

Spasticity is a consequence of neuromuscular disorders, which affects quality of life in those who experience this phenomenon. Confusion regarding spasticity and related phenomenon in clinical practice warrants a pragmatic approach to this issue. Similar terminologies for hypertonia must be evaluated in the context of pathophysiology, impact on functioning, symptom consequences, and treatment options. The goals of rehabilitation are to improve function, comfort, and ADLs, decrease caregiver burden, and prevent pressure ulcers. Clinicians working in the field of rehabilitation strive to help people make their own lives work. This requires careful assessment and intervention of spasticity related symptoms.

## References

[1] World Health Organization Disability and rehabilitation: World report on disability. http://www.who.int/disabilities/world_report/2011/report/en/index.html.

[2] National Institute of Neurological Disorders and Stroke http://www.ninds.nih.gov/disorders/spasticity/spasticity.htm.

[3] Martin A, Abogunrin S, Kurth H, Dinet J (2014). Epidemiological, humanistic, and economic burden of illness of lower limb spasticity. *Neuropsychiatric Disease and Treatment*.

[4] Lance JW, de Gail P (1965). Spread of phasic reflexes in normal and spastic subjects. *Journal of Neurology, Neurosurgery, and Psychiatry*.

[5] Lance JW (1980). The control of muscle tone, reflexes, and movement: Robert Wartenberg lecture. *Neurology*.

[6] Bhimani RH, McAlpine CP, Henly SJ (2012). Understanding spasticity from patients' perspectives over time. *Journal of Advanced Nursing*.

[7] Boyd RN, Ada L, Barnes MP, Johnson GR (2008). Physiotherapy management of spasticity. *Upper Motor Neurone Syndrome and Spasticity : Clinical Management and Neurophysiology*.

[8] Widmaeir E, Raff H, Strang K (2014). *Vanders Human Physiology: The Mechanism of Body Function*.

[9] Sanderson AB, Arnold WD, Elsheikh B, Kissal JT (2014). The clinical spectrum of isolated peripheral motor dysfunction. *Muscle & Nerve*.

[10] Bhimani R (2008). Intrathecal baclofen therapy in adults and guideline for clinical nursing care. *Journal of Rehabilitation Nursing*.

[11] Mullick AA, Musampa NK, Feldman AG, Levin MF (2013). Stretch reflex spatial threshold measure discriminates between spasticity and rigidity. *Clinical Neurophysiology*.

[12] Pati F, Vila C (2014). Symptoms, prevalence and impact of multiple sclerosis in younger patients: a multinational survey. *Neuroepidemiology*.

[13] Jušić A (2013). Differential diagnosis and treatment of muscle hypertonia as practiced in Zagreb's Centre/Institute for Neuromuscular Diseases. *Acta Myologica*.

[14] Sheean G, McGuire JR (2009). Spastic hypertonia and movement disorders: pathophysiology, clinical presentation, and quantification. *Physical Medicine & Rehabilitation*.

[15] Nielsen JB, Crone C, Hultborn H (2007). The spinal pathophysiology of spasticity—from a basic science point of view. *Acta Physiologica*.

[16] Muhkerjee A, Chakravarty A (2010). Spasticity mechanism-for the clinicians. *Frontiers in Neurology*.

[17] Burridge JH, Wood DE, Hermens HJ (2005). Theoretical and methodological considerations in the measurement of spasticity. *Disability and Rehabilitation*.

[18] Cook WA (1967). Antagonistic muscles in the production of clonus in man. *Neurology*.

[19] Beres-Jones JA, Johnson TD, Harkema SJ (2003). Clonus after human spinal cord injury cannot be attributed solely to recurrent muscle-tendon stretch. *Experimental Brain Research*.

[20] Wallace DM, Ross BH, Thomas CK (2005). Motor unit behavior during clonus. *Journal of Applied Physiology*.

[21] Boyraz I, Oktay F, Celik C, Akyuz M, Uysal H (2009). Effect of cold application and tizanidine on clonus: clinical and electrophysiological assessment. *Journal of Spinal Cord Medicine*.

[22] Bhimani RH, Anderson LC, Henly SJ, Stoddard SA (2011). Clinical measurement of limb spasticity in adults: state of the science. *Journal of Neuroscience Nursing*.

[23] Bravo-Esteban E, Taylor J, Abián-Vicén J (2013). Impact of specific symptoms of spasticity on voluntary lower limb muscle function, gait and daily activities during subacute and chronic spinal cord injury. *NeuroRehabilitation*.

[24] Silver B, Wulf SR (2014). Stroke: posthospital management and recurrence prevention. *Family Practice Essentials*.

[25] Mahoney JS, Engebretson JC, Cook KF, Hart KA, Robinson-Whelen S, Sherwood AM (2007). Spasticity experience domains in persons with spinal cord injury. *Archives of Physical Medicine and Rehabilitation*.

[26] Sanders C (2003). Application of Colaizzi's method: interpretation of an auditable decision trail by a novice researcher. *Contemporary Nurse*.

[27] Sosha GA (2012). Employment of Colaizzis strategy in descriptive phenomenology: a reflection of a researcher. *European Scientific Journal*.

[28] Lincoln YS, Guba EG (1985). *Naturalistic Inquiry*.

[29] Delhaas EM, Beersen N, Redekop WK, Klazinga NS (2008). Long-term outcomes of continuous intrathecal baclofen infusion for treatment of spasticity: a prospective multicenter follow-up study. *Neuromodulation*.

[30] McGuinness S, Hillan J, Caldwell SB (2013). Botulinum toxin in gait dysfunction due to ankle clonus: a case series. *NeuroRehabilitation*.

[31] Van Manen M (1990). *Researching Lived Experience: Human Science for an Action Sensitive Pedagogy*.

[32] Al-Araji AH, Oger J (2005). Reappraisal of Lhermitte's sign in multiple sclerosis. *Multiple Sclerosis*.

